# Treatment, prevention and public health management of impetigo, scabies, crusted scabies and fungal skin infections in endemic populations: a systematic review

**DOI:** 10.1111/tmi.13198

**Published:** 2019-01-28

**Authors:** Philippa J. May, Steven Y. C. Tong, Andrew C. Steer, Bart J. Currie, Ross M. Andrews, Jonathan R. Carapetis, Asha C. Bowen

**Affiliations:** ^1^ Northern Territory Centre for Disease Control Casuarina Australia; ^2^ Victorian Infectious Diseases Service Royal Melbourne Hospital The University of Melbourne at the Peter Doherty Institute for Infection and Immunity Parkville Australia; ^3^ Menzies School of Health Research Charles Darwin University Casuarina Australia; ^4^ Royal Children's Hospital Parkville Australia; ^5^ Murdoch Children's Research Institute University of Melbourne Parkville Australia; ^6^ Royal Darwin Hospital Casuarina Australia; ^7^ National Centre for Epidemiology & Population Health Australian National University Canberra Australia; ^8^ Perth Children's Hospital Nedlands Australia; ^9^ Wesfarmers Centre for Vaccines and Infectious Diseases University of Western Australia Nedlands Australia; ^10^ School of Medicine University of Western Australia Nedlands Australia; ^11^ University of Notre Dame Australia Fremantle Australia

**Keywords:** impetigo, scabies, crusted scabies, tinea, impétigo, gale, gale en croûte, teigne

## Abstract

We conducted a systematic review of the treatment, prevention and public health control of skin infections including impetigo, scabies, crusted scabies and tinea in resource‐limited settings where skin infections are endemic. The aim is to inform strategies, guidelines and research to improve skin health in populations that are inequitably affected by infections of the skin and the downstream consequences of these. The systematic review is reported according to the PRISMA statement. From 1759 titles identified, 81 full text studies were reviewed and key findings outlined for impetigo, scabies, crusted scabies and tinea. Improvements in primary care and public health management of skin infections will have broad and lasting impacts on overall quality of life including reductions in morbidity and mortality from sepsis, skeletal infections, kidney and heart disease.

## Abstract (fr)

Nous avons effectué une analyse systématique du traitement, de la prévention et du contrôle de santé publique des infections cutanées comprenant l'impétigo, la gale, la gale en croûte et la teigne, dans des cadres à ressources limitées où les infections cutanées sont endémiques. Le but étant d'informer les stratégies, les directives et la recherche pour améliorer la santé de la peau dans les populations qui sont touchées de manière inéquitable par les infections cutanées et leurs conséquences plus tard. La revue systématique est rapportée selon la déclaration PRISMA. Sur 1759 titres recensés, 81 études en texte intégral ont été passées en revue et les principaux résultats rapportés concernant l'impétigo, la gale, la gale en croûte et la teigne. Les améliorations apportées dans la prise en charge des infections de la peau dans les soins de santé primaires et les soins de santé publique auront des répercussions vastes et durables sur la qualité de vie en général, notamment une réduction de la morbidité et de la mortalité dues au sepsis, aux infections du squelette, aux maladies du rein et du cœur.

## Introduction

Children in developing countries and other resource‐limited settings bear a disproportionate burden of skin infections, owing to poverty, poorer living conditions, normalisation and limited access to primary healthcare 1, 2, 3, 4. More than 162 million children are estimated to have impetigo at any one time 5 and more than 110 million children with scabies 6. There are no estimates for the global burden of tinea in children, although fungal skin infections were the leading skin disease and placed in the top 10 most prevalent diseases worldwide in 2010 7.

Primary infection with impetigo and secondary bacterial infection of scabies, crusted scabies and tinea with the bacteria *Staphylococcus aureus* and *Streptococcus pyogenes* (Group A Streptococcus, GAS) lead to morbidity, mortality and socioeconomic costs via invasive infection 8, 9. Invasive *S. aureus* has a global incidence estimate of 20–50 cases/100 000 population per year with a case fatality rate of 5–30% 10, 11. An estimated 163 000 people die from GAS bacteraemia each year 8. Moreover, post‐streptococcal sequelae of acute rheumatic fever (ARF) and acute post‐streptococcal glomerulonephritis (APSGN) can lead to long‐term consequences of chronic heart and kidney disease 8, 12, 13. Due to differences in the social determinants of health, there exists a marked disparity in the burden of skin infections and their sequelae between resource‐rich and resource‐limited settings 14.

Systematic reviews of skin infection treatments that have only included randomised clinical trials (RCT) 15, 16, 17, 18, exclude a large body of available evidence from resource‐limited settings where the burden is highest 5, 6, 7. RCTs are often conducted in hospital outpatient departments (OPD) in high‐income settings, and findings may not be directly applicable to resource‐limited settings where cultural practices, access, availability, cost and acceptability of treatments may differ. There remains a lack of consensus on the best treatments and population health approaches for the prevention and control of skin infections, both individual skin conditions and skin infections collectively, in these resource‐limited settings due to a lack of a review of the evidence that is externally valid to these populations. We conducted a systematic review of studies from resource‐limited and endemic settings regarding the prevention, treatment and public health management of impetigo, scabies, crusted scabies and tinea to inform the development of evidence‐based guidelines and future research priorities for skin infections in endemic populations.

## Methods

### Search strategy and selection criteria

This systematic review is reported according to the Preferred Reporting items for Systematic Reviews and Meta‐Analyses (PRISMA) statement 19. The methods and search strategy have been described previously 20. Briefly peer reviewed and grey literature databases were searched. Studies published in English since 1960 using any experimental study (RCTs, clinical controlled trials, before and after studies and interrupted time series analyses) or observational study design (cohort and ecological studies) were included. Eligible participant types included Indigenous peoples and populations in resource‐limited settings (low, low‐middle and middle‐income countries and resource‐limited populations in Organisation for Economic Co‐operation and Development (OECD) countries) (see Appendix S1 for definitions) with a diagnosis of impetigo, scabies, crusted scabies, tinea capitis, tinea corporis or tinea unguium (onychomycosis) in persons of any age or sex. We reviewed any clinical or public health interventions aiming to reduce skin infections with any type of comparator. Outcomes were categorised as primary (cure or decrease in prevalence for population‐based studies) or secondary (microbiological cure, symptom relief, recurrence, adherence, acceptability, adverse events and spread to contacts). Two authors (AB and PM) independently screened the titles and abstracts of all studies identified in the search process and selected the studies for eligibility assessment. Full reports of these studies were obtained and assessed by two independent reviewers (10 reviewers in total). Any discrepancies for inclusion were resolved by consensus discussion.

### Assessment of methodological quality and data extraction

Two reviewers independently scored for methodological quality of clinical trials using *The Cochrane Collaboration's tool for assessing risk of bias* 21. Observational studies were assessed for blinding, completeness of outcome data, outcome reporting and other sources of bias including confounders. All data were entered into data extraction forms using Covidence online software (Veritas Health Innovation, Melbourne, VIC, Australia) by the two independent reviewers and discrepancies resolved via discussion.

### Statistical analysis and synthesis

The data are presented in a narrative synthesis. Meta‐analysis was not performed due to the heterogeneity of studies. Calculations were performed using STATA13 (Statacorp, Texas, USA). For reading ease, results are presented in common theme groups in each area of clinical treatment or public health prevention and control relevant to skin infections in resource‐limited settings. As many population‐based studies incorporate multiple strategies such as health education, treatment and hygiene practices, it is recommended that all evidence is considered by the reader as a whole. We used the GRADE approach to rate evidence across studies for specific clinical outcomes to link evidence‐quality evaluations to recommendations in clinical guidelines (Table 1).

**Table 1:** Grading of recommendations assessment, development and evaluation evidence grades and strength of recommendations

Code	Quality of evidence	Definition
A	High	Further research is very unlikely to change the level of confidence in the estimate of effect. i.e. Several high‐quality studies with consistent results
B	Moderate	Further research is likely to have an impact in current confidence in the estimate of effect and may change the estimate. i.e. One high‐quality study Several studies with some limitations
C	Low	Further research is very likely to have an important impact on the level of confidence in the estimate of effect and would likely change the estimate. i.e. One or more studies with severe limitations
D	Very Low	Estimate of effect is very uncertain. i.e. No direct research evidence One of more studies with very severe limitations

Code	Strength of recommendation	Implications when combined with evidence grade
1	Strong	1A: Strong recommendation, applies to most patients without reservation. Clinicians should follow a strong recommendation unless a clear and compelling rationale for an alternative approach is present. 1B: Strong recommendation, applies to most patients. Clinicians should follow a strong recommendation unless a clear and compelling rationale for an alternative approach is present. 1C: Strong recommendation, applies to most patients. Some of the evidence base supporting the recommendation is, however, of low quality. 1D: a Strong recommendation, applies to most patients. However, the recommendation is based on expert consensus only.
2	Weak	2A: Weak recommendation and best action may differ depending on circumstances or patients or societal values.
		2B: Weak recommendation and alternative approaches likely to be better for some patients under some circumstances. 2C: Very weak recommendation; other alternatives may be equally reasonable. 2D: a Very weak recommendation based on expert consensus. Further research is necessary.

a 1D and 2D recommendations are not routinely included by the GRADE approach as these are based on expert consensus, rather than scientific evidence. These additional recommendation grades were created due to lack of available supporting evidence but an identified need to make recommendations to guide clinical and public health management.

## Results

**Figure 1:**
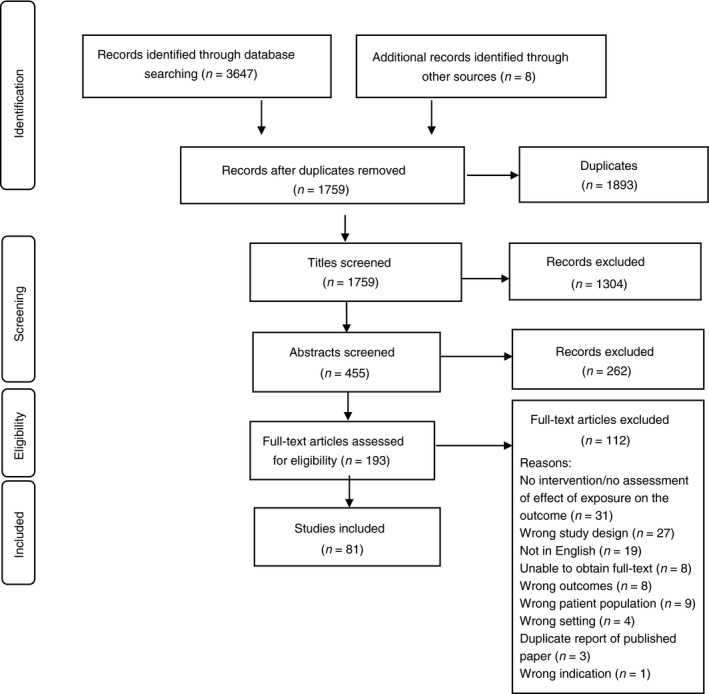
PRISMA flow diagram for study selection in the systematic review.

**Figure 2:**
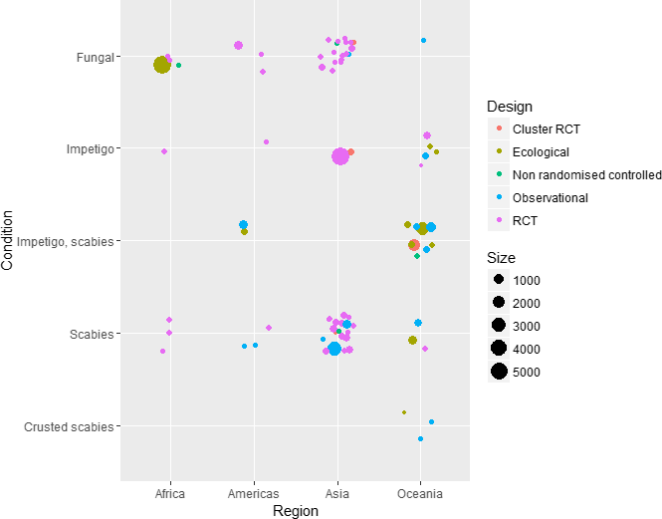
Selected summary characteristics of studies included in the systematic review. [Colour figure can be viewed at http://wileyonlinelibrary.com]

The search strategy identified 1759 titles and 455 abstracts for screening, of which 193 met the inclusion criteria. 81 full text studies were included (Figure 1), representing >27 633 participants over a 40‐year period (1976–2015). The study size, type, location and condition under study are summarised (Figure 2, Table S1). The study details and characteristics are summarised in Table 2. There were 44 (54%) RCTs, four (5%) cluster RCTs, three (4%) controlled clinical trials, three (4%) controlled before and after studies and three (4%) controlled population studies (Table S2). There were two (3%) before and after studies, four (5%) ecological studies, 14 (17%) prospective cohort studies and four (5%) retrospective observational studies appraised (Table S3).

**Table 2:** Number of studies in each broad intervention group by skin condition. Gaps in the evidence are shown as grey boxes

Intervention	Condition						Total number of studies
	Impetigo	Scabies	Scabies and impetigo	Crusted scabies	Fungal skin infections	Multiple skin conditions under study or ‘skin infections’ that were not otherwise specified	
Comprehensive community skin health programmes		1	1			1	3
Coordinated case management				1			1
Standard treatment protocols				1			1
Directed clinical treatment	4	19		1	23		47
Mass Drug Administration		4	9				13
Complimentary/alternative therapy		2			1	1	4
Communicable disease control^a^		3					3
Hygiene practices^c^	2				1		3
Water provision^b^	1		1			2	4
Housing programmes						2	2
Total	7	29	11	3	25	6	81

a Communicable disease control activities= outbreak response or treatment of contacts.

b Water provision = swimming pools or clean water supply to homes.

c Hygiene practices = provision of soap and hand‐washing education.

### Summary of clinical treatment recommendations for resource‐limited settings

#### Comprehensive community skin health programmes

Moderate quality evidence that treatment combined with comprehensive skin control measures (health promotion, environmental interventions and screening) add benefit in sustaining a reduction in scabies prevalence alone (**2B**) 22 and impetigo and scabies prevalence combined (**2C**) (Appendix S2) 23, 24, 25. No studies assessed the effect of a community skin health programme on impetigo or tinea alone, whilst one study described this for scabies 22, one for scabies and impetigo 23, 24, and one for general skin infections 25. High‐quality evidence from studies using control communities would be advantageous in determining the measurable benefit over standard treatment (Table 3).

**Table 3:** Number of included studies with public health co‐interventions for skin infections

Skin condition	Public health co‐interventions	Treatment of contacts	Promotion of regular bathing and/or hand‐washing	Health education	Washing of clothing and bed linen	Storage of items in plastic bags	Exposing items to direct sunlight	Household spraying
Impetigo	2		2	1				
Scabies	22	14	2	6	12	2	4	1
Scabies and impetigo	5	1	1	3	2		1	1
Crusted scabies								
Fungal skin infections	1		1					
General skin infections	4		2	2				
Total	34	15	8	12	14	2	5	2

Total does not equate to 81 studies as some studies had more than one public health intervention.

Grey shades represent missing data i.e., nothing known for these categories.

In Bangladesh, moderate quality evidence was provided from a study where permethrin Mass Drug Administration (MDA) was followed by randomisation of male boarding school students to a scabies control programme (repeat permethrin treatment for scabies, health promotion activities with a designated scabies class monitor, daily bathing with soap and bags for bedding and clothing storage) or control 22. At 4 months, scabies prevalence was 5% (intervention) and 50% (control), *P* < 0.001 22. In Australia, low‐quality evidence was provided from a permethrin MDA that included a comprehensive skin control programme (annual treatment and community clean up days, health promotion and repeat treatment with permethrin for scabies) in a remote Indigenous community 23, 24. Scabies prevalence declined from 35% to 12%, *P* < 0.0001 and impetigo from 11% to 2%, *P* = 0.0005 23, 24. In Kenya, low‐quality evidence was provided from a 5‐year dermatology project within primary healthcare (training of healthcare workers and school‐based treatments) that did not show a sustained reduction in impetigo, scabies or tinea 25.

#### Impetigo

##### Directed antimicrobial therapy

High‐quality evidence supports the use of oral co‐trimoxazole or intramuscular (IM) benzathine penicillin G (BPG) for the treatment of impetigo (**1A**) 26, 27. Oral amoxicillin or oral erythromycin are suitable alternatives (**2B**) 28. Oral penicillin G is not recommended for treatment of impetigo (**2D**) 29. Although topical antibiotics are recommended as the preferred treatment for impetigo in industrialised settings 16, there is no available evidence from resource‐limited contexts for topical antibiotics or evidence to not treat impetigo.

High‐quality evidence from two open label RCTs with Australian Indigenous children compared oral co‐trimoxazole *vs*. IM BPG and found no difference in clinical or microbiological cure of impetigo 26, 27. Moderate quality RCT evidence reported clinical cure in 89% of patients in both groups when oral amoxicillin and oral erythromycin for 7 days in Mali were compared 28. Low‐quality RCT evidence in Canadian Indigenous children compared oral penicillin G for 10 days with IM BPG, with treatment failure equivalent: 16% and 14% respectively 29. No studies assessed topical agents or used a placebo‐controlled design for impetigo.

##### Mass Drug Administration

No studies assessed MDA for impetigo alone. Impetigo was a secondary outcome in scabies MDAs reported below.

##### Complimentary/alternative therapies

No studies assessed complimentary therapies for impetigo.

##### Hand‐washing and hygiene practices

High‐quality evidence supports daily hand‐washing with soap for the treatment and prevention of impetigo, with no benefit found for antibacterial soap over regular soap (**1A**) 30, 31.

In Pakistan, high‐quality evidence from two RCTs enrolling households with children assessed hand‐washing with soap for impetigo and found a benefit for soap, but no difference between antibacterial (triclocarbon 1.2%) and standard soap 30, 31.

#### Scabies

All studies on scabies treatment used clinical cure or symptom relief as end points.

##### Directed anti‐parasitic therapy

###### Topical treatment *vs*. topical treatment

Seven studies compared topical anti‐parasitic agents for scabies 32, 33, 34, 35, 36, 37, 38, with low to moderate quality evidence for either topical permethrin or topical ivermectin (**2B**) 32, 33. Permethrin is superior to lindane (**1A**) 34, topical crotamiton (**2C**)^35^ or Tenutex emulsion (disulphiram and benzyl benzoate [BB]) in those >4 years (**2C**) 36. Topical ivermectin is superior to topical crotamiton in those >2 years (**2C**) 37. Very low‐quality evidence from one study shows that topical BB or topical permethrin is safe in pregnant women (**2C**) 38. Without high‐quality evidence to support modified applications of topical treatments for scabies, the standard whole‐body application remains strongly recommended (**1D**).

High‐quality RCT evidence from an Iranian hospital OPD found that two applications of 5% permethrin achieved a superior clinical cure (85%) compared to 1% lindane (49%), *P* < 0.05 34. Clinical cure was similar with topical ivermectin or topical permethrin in an Iranian dermatology OPD 33. When topical ivermectin 1% and topical permethrin 5% were compared with oral ivermectin, clinical response at 1 week was superior with either topical treatment (69% and 75% *vs*. 30%, *P* < 0.05) whilst cure at 4 weeks was universal for all three agents 32. Topical permethrin 35 and topical ivermectin 37 were superior to topical crotamiton at 4 weeks follow‐up. Topical permethrin was superior to Tenutex emulsion 36. Very low‐quality evidence from a refugee camp on the Thai‐Burmese border assessed safety of permethrin and BB in pregnancy 38.

###### Modified application of permethrin

Practice point Box: How is it best to apply topical scabicides?

Twenty‐nine studies incorporated a topical scabicide/s, mostly permethrin. (Table S4). One study directly compared neck to toe application (head to toe in children) with application to lesions only 39. Overall, head to toe or neck to toe was recommended in 26 studies, lesion only in four (three of which were topical ivermectin and not specified in seven studies. Full body application of topical scabicides is recommended (1D). The effective application of topical scabicides requires a private setting where the clothes can be removed for application. This is not always practical or achievable in overcrowded households and may limit the effect of topical therapy.

###### Oral treatment *vs*. topical treatment

Moderate to high‐quality evidence supports the use of oral ivermectin or topical permethrin for the treatment of scabies (**1A**) 32, 40, 41, 42.

A comparison of topical 5% permethrin with oral ivermectin in a high‐quality RCT from India found lesion count and pruritus significantly lower for permethrin at 1 week whilst clinical cure at 4 weeks was the same 40. Moderate quality evidence from India reached similar conclusions 32. From Iran, low‐quality evidence is provided from two studies that compared oral ivermectin with topical permethrin and found superior symptom relief with permethrin at 2 weeks, whilst clinical cure was the same 41, 42. There is moderate‐high‐quality evidence that oral ivermectin achieved superior clinical cure than topical lindane 43, 44, 45 or topical sulphur 46. Comparisons of oral ivermectin with topical BB showed discrepant results: no difference in clinical cure based on high‐quality RCT evidence from Vanuatu 47 whilst oral ivermectin was superior for clinical cure in moderate quality evidence from Senegal 48 and Nigeria 49.

##### Mass drug administration

There is moderate quality evidence for MDA to control scabies in resource‐limited communities (**1B**) 50, 51, 52, 53, with high‐quality comparison RCTs needed to determine the best agent. Moderate quality evidence for the population effect of MDA for scabies on scabies and impetigo prevalence was achieved using either topical permethrin or oral ivermectin (**1B**) 23, 54, 55, 56. Oral ivermectin is superior to topical permethrin and standard of care for community‐wide use in children >5 years and non‐pregnant adults in isolated settings with high prevalence of scabies and impetigo (**1B**) 57. High‐quality studies conducted in mainland populations are required to determine the effectiveness of the MDA approach in highly mobile populations.

###### Scabies only

Low to moderate quality evidence from four studies in Fiji 50, India 52, 53 and Tanzania 51 assessed MDA impact on scabies prevalence only. Two doses of oral ivermectin achieved a 95% reduction in scabies in India 52 whilst single dose ivermectin MDA was not superior to BB in Fiji 50. Ivermectin delivered in a lymphatic filariasis MDA reported a 68–98% decline in scabies 51. When 25% BB was delivered in an MDA to an Indian orphanage, cure was 100% at 6 weeks 53.

###### Scabies and impetigo

####### Permethrin MDA

Low‐quality evidence is provided from permethrin MDA's, which were all ecological in design with different populations reviewed at baseline and follow‐up. Four studies from Panama 56 and remote Australian Aboriginal communities 23, 54, 55 showed a reduction in scabies and impetigo prevalence following MDA with 5% permethrin. The first scabies MDA used permethrin in a remote Kuna Indian population in Panama in 1986 and although interrupted by political tensions demonstrated a sustained response 56. The permethrin MDAs were combined with impetigo treatment and broad‐based community skin programmes including surveillance, health promotion, home cleaning and retreatment of cases in Australia 23, 54, 55.

####### Ivermectin *vs*. Permethrin MDA

Moderate quality evidence is provided from a cluster RCT where oral ivermectin and topical permethrin MDAs were compared with standard case treatment with topical permethrin for scabies in three Fijian island communities 57. Ivermectin was superior at 12 months for scabies and impetigo 57.

####### Ivermectin MDA

Low‐quality evidence is provided from two studies that assessed the effect of oral ivermectin MDA on scabies prevalence 58, 59. In the Solomon Islands, two doses of oral ivermectin reduced the prevalence of scabies at 3 years 59 and this was sustained at a further follow‐up 15 years later 60. In contrast, an oral ivermectin MDA delivered in a remote Australian Aboriginal community did not show significant or sustained declines in scabies prevalence 58.

####### Azithromycin MDA

Very low‐quality evidence from an azithromycin MDA for trachoma in a remote Australian Aboriginal population reported impetigo reduction at 2–3 weeks which returned to baseline at 6 months 61. Scabies prevalence was unchanged 61.

##### Complimentary therapy

Moderate quality evidence that cold cream can be used as an adjunct to topical sulphur for scabies (**2B**) 62 .

In a Mexican orphanage RCT, topical 10% sulphur in pork fat was compared with topical 10% sulphur in cold cream with high rates of cure 62. Preliminary data for aloe vera for scabies treatment 63.

##### Communicable disease control and prevention

There is low‐quality evidence for treatment of household contacts for the community control of scabies (**2C**) 64. Treatment of cases and contacts is recommended in scabies outbreaks (**2C**), however, high‐quality studies comparing treatments during outbreaks are required.

Low‐quality evidence for the treatment of household contacts as the primary intervention for scabies control from one cohort of Australian Aboriginal households where a sixfold reduction in scabies in compliant households was found 64. Fifteen other studies treated close contacts, family members or the household as co‐interventions for scabies, however, without a comparison group, the effect cannot be reliably assessed. Moderate quality evidence found that oral ivermectin halted a scabies outbreak amongst healthcare workers and patients in Peru 65, and topical BB for cases and contacts with community education terminated an outbreak in Israel 66.

##### Environmental co‐interventions

Although washing and storage measures are unlikely to cause harm and should be encouraged, high‐quality studies assessing the clinical effectiveness of washing clothing and bed linen, storage of items in plastic bags, exposure to sunlight and household spraying are required before these measures can be strongly recommended as adjuncts in the control of scabies. No studies used a control group to assess the effect of environmental interventions for scabies. Twelve studies included washing of clothing and bed linen 34, 39, 40, 43, 47, 48, 49, 50, 53, 62, 63, 64, 65, two studies included storage of items in plastic bags 22, 65, four studies included exposing items to direct sunlight 39, 47, 49, 53 and one study included household spraying 66, as co‐interventions (Table 2).

#### Crusted scabies

Moderate quality evidence supports oral ivermectin with topical keratolytics and topical antiparasitics for crusted scabies (**1B**) 67, 68. Comparative trials are needed to explore more effective treatments. Patients with crusted scabies require intensive supportive treatment (**1B**) 67, 68. Coordinated case management in the home may be of benefit (**2C**) 69.

##### Directed antimicrobial therapy

Moderate quality evidence from a prospective cohort study of Australian Aboriginal inpatients receiving oral ivermectin at days 0, 14 and 28 and daily topical permethrin alternating with keratolytic therapy (topical urea 10% and lactic acid 5%), found 40% achieved complete cure at 4 weeks 68.

##### Standard treatment protocols

Moderate‐quality evidence from a retrospective study used a standard treatment protocol in Australian Aboriginal inpatients with crusted scabies achieving 55% without recurrence at 8 years 67.

##### Coordinated case management

Low‐quality evidence supports topical BB, regular keratolytics, moisturiser and regular screening for new lesions in home‐based case management to prevent crusted scabies 69.

#### Fungal skin infections

##### Directed antimicrobial therapy

###### Tinea capitis

Moderate quality evidence for griseofulvin, terbinafine and fluconazole having similar efficacy for tinea capitis (**1B**) 69, 70, 71, 72, 73. Tinea capitis is difficult to treat, takes several months and mycological cure is challenging.

High‐quality evidence of similar clinical and mycological cure was provided by a multicentre RCT from Guatemala, Chile, Costa Rica, USA and India comparing daily oral fluconazole for 3 or 6 weeks with daily griseofulvin 70. Low‐quality RCT evidence from Iran reported no difference between daily fluconazole or daily griseofulvin at 8 weeks 71. Low‐quality evidence from India found griseofulvin twice daily, fluconazole weekly and terbinafine daily all performed similarly 72. In addition, all used ketoconazole 2% shampoo and prednisolone prescribed for kerion 72. From China, low‐quality cluster RCT evidence confirmed griseofulvin daily for 4 weeks or terbinafine daily for 2–4 weeks performed similarly 73.

###### Tinea corporis

Low to moderate quality evidence for topical sertaconazole, butenafine, miconazole or clotrimazole over other agents for tinea corporis (**2C**) 74, 75, 76, 77. Low‐quality evidence that oral alternatives for tinea corporis are terbinafine or fluconazole (**2C**) 78. Although the systematic review on topical treatments for tinea corporis recommends topical terbinafine as a first‐line agent 17, no high‐quality studies from resource‐limited contexts were available to evaluate. Most included trials came from dermatology outpatient clinics in India or Iran. Community setting, population level evidence is needed for tinea corporis treatment.

Moderate quality RCT evidence from Iran confirmed similar clinical cure at 8 weeks for topical butenafine compared with topical clotrimazole 76 and similar cure rates at 4 weeks for topical miconazole and topical sertaconazole 74. Moderate quality RCT evidence from India found sertaconazole outperformed miconazole with 62% and 45% cured at 2 weeks respectively, *P* < 0.05 75. Low‐quality evidence from India found topical clotrimazole and topical amorolfine were comparable 79 and that topical sertaconazole was superior to topical butenafine 77. Similarly, very low‐quality pilot RCT evidence from India found superiority of topical sertaconazole over topical terbinafine or topical luliconazole for clinical cure and symptom relief 80. Very low‐quality RCT evidence also found no difference between topical sertaconazole and topical terbinafine 81 and that topical terbinafine and topical luliconazole could not be differentiated 82. Similarly, low‐quality RCT evidence from India found that daily oral terbinafine or weekly fluconazole achieved similar clinical cures 78 and topical butenafine was no better than weekly fluconazole combined with topical Whitfield's ointment (3% salicylic acid and 6% benzoic acid) at 4 weeks 83. Low‐quality evidence from a prospective cohort of Australian Aboriginal people with tinea corporis and tinea unguium found daily oral terbinafine cured 32% 84.

###### Tinea unguium/onychomycosis

For tinea unguium, moderate to high‐quality evidence recommends oral terbinafine (**1A**) 85, 86, 87, with no added benefit of combination topical therapy in resource‐limited settings (**1B**) 85, 88. Surgical avulsion prior to treatment of onychomycosis is not recommended (**2D**) 88. High‐quality studies assessing photodynamic therapy (PDT) regimens for tinea unguium are required to determine the utility of this therapy in resource‐limited settings.

High‐quality RCT evidence from India trialled two different dosing regimens of terbinafine and showed no difference 85, 86. Low‐quality RCT evidence from Brazil found monthly or second monthly dosing of oral terbinafine had similar outcomes 87 and photodynamic therapy (PDT) every 15 days for 6 months was superior to weekly oral fluconazole 89. No additional benefit of topical nail lacquer over oral terbinafine alone was found in moderate quality evidence 85, 88.

##### Mass drug administration

No studies assessed the effect of antifungal MDAs on the prevalence of fungal skin infections.

##### Complimentary/alternative therapy

Further studies are needed to assess the role of aloe vera gel, as only very low‐quality evidence from one study is available 90.

##### Communicable disease prevention and control

No studies assessed the effect of communicable disease control practices on fungal infections on which to base relevant recommendations for resource‐limited settings.

##### Hygiene practices

Daily soap use may be of benefit in the treatment of tinea capitis and tinea corporis. This is recommended in combination with anti‐fungal treatment (**2C**) 91.

From Tanzania, low‐quality RCT evidence found mycological cure at 2 months to be similar with either daily washing with triclosan soap or placebo 91.

#### Infrastructure including high‐quality water supply, swimming pools and housing improvement for skin infections

##### Water provision

An adequate supply of water for washing and cleaning will reduce the burden of impetigo and scabies (**2C**) 92. From studies in remote Australian Indigenous communities, the installation of community swimming pools may assist in the prevention of impetigo, along with other health benefits (**2C**) 93, 94, 95. No studies assessed the effect of quality water supply or swimming pools on scabies or tinea on which to base recommendations for resource‐limited settings.

Low‐quality evidence from Panama found that when unlimited, high‐quality water was compared to a community with a limited water supply, declines in scabies and impetigo incidence were reported 92. Low‐quality evidence from three studies in Australian Aboriginal communities found a small benefit following the installation of swimming pools for impetigo and skin infections 93, 94, 95.

##### Housing improvement programmes

Programmes to improve housing may assist in the prevention and control of skin infections in resource‐limited populations (**2C**) 96, 97.

Low‐quality evidence from a housing intervention evaluation of remote Australian Aboriginal communities, found construction of new, standardised housing and the demolition of uninhabitable dwellings did not change the prevalence of skin infections at 10 months 96. Low‐quality evidence from a study that ran for 12 years showed reductions in skin infections following household improvements based on health and safety priorities in a ‘survey and fix methodology.’^97^

## Discussion

This is the first systematic review to comprehensively inform treatment, public health control and areas for future research in the control of skin infections using evidence generated in and from settings where skin infection burden is the highest. High‐quality evidence for treatment of the individual and community with scabies and for the individual with impetigo is synthesised for inclusion into evidence‐based guidelines. Similarly, high‐quality evidence for comprehensively addressing scabies and impetigo concurrently is presented, with further studies needed to determine the measurable benefit of additional interventions over treatment alone. The integration of oral antibiotics for treatment of impetigo, use of oral ivermectin or topical permethrin MDA for scabies in endemic or outbreak settings and community education and health promotion activities in skin health programmes are supported by the evidence and should form the basis of skin control programmes when needed. Evidence gaps include community control of dermatophyte infections and targeted environmental health interventions to improve skin health.

Progress towards the streamlined integration of data collection on skin infections when planning MDAs for other infections needs ongoing prioritisation. MDA for trachoma and yaws with azithromycin 98, 99, 100 may also reduce the burden of impetigo 61, whilst ivermectin MDA for lymphatic filariasis 101 and scabies 57 will reduce scabies and impetigo prevalence 61 as part of the roadmap towards defeating neglected tropical diseases 102. This pragmatic, evidence‐based strategy is now being tested in larger populations with results awaited (ACTRN12618000461291p) to inform whether community control of scabies will prevent severe skin infections.

For impetigo, duration of treatment, the role of topical therapy and added benefit of comprehensive skin disease control programmes over treatment alone are gaps in the literature. Whilst 3 or 5 days of cotrimoxazole for impetigo treatment in resource‐limited settings is effective 26, more comparison studies are needed to optimise treatment duration and utility of cheap, widely available, palatable alternative agents in high‐burden contexts. Cephalexin for up to 10 days remains in guidelines for impetigo, yet this is lengthy, costly and may be impractical with no evidence supporting its use for impetigo in high‐burden contexts. Unlike developed settings where topical mupirocin and fusidic acid are recommended 16, there are currently no trials using topical antibiotics for impetigo in high burden settings. Results from New Zealand comparing topical antibiotics or antiseptics with placebo are awaited [ACTRN1261000356460].

Knowledge gaps identified include the patient preference for agent to treat scabies, and the additional benefit of comprehensive control programmes for scabies above treatment alone. Topical permethrin has more rapid reduction in symptoms 40, 42 but requires a private space in which to apply the cream to the full body. Conversely, clinical response is slower, but ease of administration and overall community efficacy in MDA support the use of ivermectin 57. Future studies should address the role of a second dose of ivermectin in asymptomatic individuals as unhatched eggs are refractory to ivermectin 103. Moxidectin shows promise for future human scabies trials as it has a longer half‐life and is ovicidal 104.

Most studies assessing antifungal treatments were from dermatology OPD in middle‐income country hospitals, which limits the external validity to other resource‐limited settings. Studies assessing the effectiveness of topical and oral (for severe disease) treatments of tinea in a range of resource‐limited populations would be of benefit to make recommendations applicable to real life and uncontrolled settings at the individual and population level. Future integration of treatment of tinea into comprehensive skin disease control programmes that address scabies and impetigo may be a way forward.

Despite practical advantages, we found limited evidence for environmental interventions to control skin infections. Although sound attempts to evaluate housing programmes have been made 96, 97, we remain unable to recommend small‐scale environmental interventions due to a lack of comparative studies. For example, no studies compared household spraying with no intervention to eradicate the scabies mite. Similarly, there was no evidence for hot washing of clothing compared to not washing clothing. Although environmental measures are unlikely to cause harm in combination with treatment of the skin infection, research is needed to determine any measurable benefit above standard treatment to inform environmental health teams tasked with managing scabies outbreaks, clinicians managing skin infections or governments and communities intending to include environmental policy recommendations in comprehensive skin health programmes in endemic areas.

Although 1759 non‐duplicate studies were found for potential inclusion in this systematic review, most were excluded prior to the final appraisal of 81 studies meeting the full inclusion criteria (see Figure 1). This is the complete synthesis of available literature on these four skin conditions. It is possible that restriction to English language publications or being unable to find the full text publication has been a limitation in the scope of this, although <30 full‐text studies were excluded for this reason.

## Conclusions

A summary of the evidence‐based recommendations for skin infections in high‐burden contexts also highlights the need for further rigorous, experimental studies to fill the evidence gaps. Pragmatic, practical, high‐quality, well‐funded RCTs are essential in the settings where the findings will have external validity if meaningful progress is to be made towards reducing the gap in skin health outcomes between the rich and poor. Acknowledging that RCTs may present ethical issues for some groups 105, robust observational studies of appropriately funded public health interventions can be tested across large populations with designs that control for confounders and in meaningful partnership with the communities under study using participatory research methods.

## Supporting information


**Table S1.** List of studies included in the systematic review.
**Table S2.** Risk of bias table with overall quality ratings using the GRADE approach for included experimental and controlled studies
**Table S3.** Risk of bias table with overall quality rating using the GRADE approach for included observational studies
**Table S4.** Method of application of topical scabicides in 29 included studies
**Appendix S1.** Definitions for Indigenous peoples and Income groupings used
**Appendix S2.** Evidence Summary and Recommendations for skin infection‐related research to guide practice in resource‐limited settings.
**Data S1.** PRISMA Checklist
