# Supplementary material for: Treatment, prevention and public health management of impetigo, scabies, crusted scabies and fungal skin infections in endemic populations: a systematic review
Source: Trop Med Int Health. 2019 Jan 28;24(3):280–93. doi: 10.1111/tmi.13198 (PMC6850630; doi:10.1111/tmi.13198)
Supplement: Supplementary file 1 — Table S1. List of studies included in the systematic review. Table S2. Risk of bias table with overall quality ratings using the GRADE approach for included experimental and controlled studies Table S3. Risk of bias table with overall quality rating using the GRADE approach for included observational studies Table S4. Method of application of topical scabicides in 29 included studies Appendix S1. Definitions for Indigenous peoples and Income groupings used Appendix S2. Evidence Summary and Recommendations for skin infection‐related research to guide practice in resource‐limited settings. Data S1. PRISMA Checklist [file TMI-24-280-s001.docx]

**Supplementary Appendix S1**

**Definitions Box**

Indigenous peoples: identify as being part of a distinct cultural group who maintain this identity and customs separate from the dominant culture. Indigenous people are descended from groups present within a region before current states and borders were defined.

Income groupings: All 189 World Bank member countries, plus 28 other economies with populations > 30,000, are classified by gross national income (GNI) per capita, in U.S. dollars in 2016, into four income groupings: low (<= $1,045), low-middle ($1,046-4,125), upper-middle ($4,126-12,735) and OECD (> $12,735). (<https://datahelpdesk.worldbank.org/knowledgebase/articles/906519-world-bank-country-and-lending-groups>, last accessed April 2018)

**Box:** Definitions for Indigenous peoples and Income groupings used

**Appendix S2: Evidence Summary and Recommendations for skin infection related research to guide practice in resource limited settings**

1. Comprehensive Community Skin Health Programs
   1. Treatment combined with comprehensive skin control measures (health promotion, environmental interventions and screening) add benefit in sustaining a reduction in scabies prevalence alone (**GRADE 2B**)^22^ and impetigo and scabies prevalence combined (**GRADE 2C**).^23-25^
   2. Further studies are required to determine whether there is any difference between the provision of these comprehensive measures and simply additional applications of topical permethrin, additional doses of oral ivermectin or the need for directed impetigo treatment.
   3. High quality studies using control communities who do not receive the additional interventions would be advantageous in determining the measurable benefit over standard treatment alone.
2. Impetigo
   1. Directed Antimicrobial Therapy:
      1. High quality evidence for impetigo treatment: oral co-trimoxazole or intramuscular (IM) benzathine penicillin G (BPG) (**GRADE 1A**).^26,27^ Co-trimoxazole has fewer side effects.
      2. Moderate quality evidence for impetigo treatment: oral amoxicillin or oral erythromycin are suitable alternatives (**GRADE 2B**).^28^ Amoxicillin has fewer side effects.
      3. Oral penicillin G is not recommended for impetigo treatment due to limited low quality evidence (**GRADE 2D**).^29^
      4. There is no evidence to support the use of topical antibiotics or for no treatment of impetigo.
   2. Mass Drug Administration (MDA):
      1. There is no evidence to support MDA for impetigo alone. Further research is needed, with reporting of changes in impetigo prevalence in azithromycin MDAs for yaws and trachoma encouraged.
   3. Complimentary/alternative therapies:
      1. There is no evidence to support the use of complimentary therapies for impetigo.
   4. Handwashing and hygiene practices:
      1. High quality evidence for once daily washing with soap for the treatment and prevention of impetigo (**GRADE 1A**).^30,31^
      2. No benefit to the use of antibacterial soap over regular soap for impetigo (**GRADE 1A**).^30,31^
3. Scabies
   1. Directed anti-parasitic therapy
      1. Moderate to high quality evidence for topical permethrin or oral ivemectin for scabies treatment (**GRADE 1A**).^32,40-42^ Permethrin provides a faster clinical response and earlier symptom relief.
      2. Low to moderate quality evidence for topical permethrin or topical ivermectin in scabies treatment (**GRADE 2B**).^32,33^
      3. High quality evidence for permethrin above lindane for scabies treatment (**GRADE 1A**).^34^
      4. Low quality evidence for permethrin above crotamiton or tenutex emulsion in people aged over four years (**GRADE 2C**).^35,36^
      5. Low quality evidence for topical ivermectin above crotamiton in people aged over two years (**GRADE 2C**).^37^
      6. Low quality evidence that benzyl benzoate or permethrin is safe for scabies treatment in pregnant women (**GRADE 2C**).^38^
      7. The standard application of topical scabies treatments covering all body surfaces remains strongly recommended (**GRADE 1D**).**^39^**
   2. Mass Drug Administration (MDA):
      1. Moderate quality evidence for oral ivermectin or topical permethrin MDA to control scabies and associated impetigo (**GRADE 1B**).^23,54-57^
      2. Moderate quality evidence for superiority of oral ivermectin for community-wide use in children>5 years and non-pregnant adults (**GRADE 1B**).^57^
      3. High quality comparative studies are needed to determine the best agent for MDA and report on the community preferences associated with different treatment options.
   3. Complimentary Therapy:
      1. Moderate quality evidence for cold cream as an adjunct to topical sulphur therapy for scabies (**GRADE 2B**).^62^
   4. Communicable Disease Control and prevention:
      1. Low quality evidence for treatment of household contacts for the community control of scabies (**GRADE 2C**).^63^
      2. Treatment of cases and contacts is recommended in scabies outbreaks (**GRADE 2C**).^63^
   5. Environmental co-interventions:
      1. Although these practices are unlikely to cause harm and have been included in a range of studies, there is no evidence assessing the clinical effectiveness of washing clothing and bed linen, storage of items in plastic bags, exposure to sunlight and household spraying as adjuncts in the control of scabies alone.
4. Crusted scabies
   1. Treatment:
      1. Moderate quality evidence for oral ivermectin with topical keratolytics and topical antiparasitics for crusted scabies treatment (**GRADE 1B**).^66,67^
      2. Further comparative trials in resource-limited populations would be beneficial to explore more effective treatments.
   2. Prevention:
      1. Moderate quality evidence that patients with crusted scabies in resource-limited settings require intensive supportive treatment (**GRADE 1B**).^66,67^
      2. Low quality evidence that coordinated case management in the home may be of benefit (**GRADE 2C**).^68^
5. Fungal skin infections
6. Directed antimicrobial therapy
   - 1. Tinea capitis:
        1. Moderate quality evidence that oral griseofulvin and oral fluconazole have similar efficacy (**GRADE 1B**).^69-72^ Affordability and availability influence first line treatment recommendations.
     2. Tinea corporis:
        1. Topical Treatment: Low to moderate quality evidence for topical sertaconazole, topical butenafine, topical miconazole or topical clotrimazole over other agents (**GRADE 2C**).^73-76^
        2. Oral Treatment: Low quality evidence for treatment with oral terbinafine or oral fluconazole (**GRADE 2C**).^84^
        3. Additional studies of antifungal treatments for tinea corporis conducted in community settings would be beneficial in assessing the effectiveness of treatment at the population level in resource-limited settings.
     3. Tinea unguium (onychomycosis):
        1. Moderate to high quality evidence for treatment with oral terbinafine (**GRADE 1A**).^84-86^
        2. Moderate quality evidence of no added benefit for combinations of topical therapy and oral therapy (**GRADE 1B**).^84,87^
        3. Low quality evidence that surgical avulsion of the nail prior to treatment is not recommended (**GRADE 2D**).^87^
        4. High quality studies assessing photodynamic therapy (PDT) regimens are required to determine the utility of this therapy in resource-limited settings.
7. Comprehensive community skin health programs: Robust trials assessing the effect of skin health programs, coordinated case management, mass drug administration and standardised treatment protocols targeting fungal infections are required.
8. Complimentary/alternative therapy:
   1. RCTs comparing aloe vera gel with standard treatments are needed before aloe vera gel can be recommended.
9. Communicable disease prevention and control:
   1. Robust trials to determine the utility of disease prevention and control activities for fungal infections in endemic populations are required.
10. Hygiene practices:
11. Daily soap use may be of benefit in the treatment of tinea capitis and tinea corporis, however, due to low quality evidence this is recommended in combination with anti-fungal treatment (**GRADE 2C**).^90^
12. There is no evidence to support any added benefit of triclosan soap over normal soap in resource-limited settings.

6. Infrastructure including high quality water supply, swimming pools and housing improvement for skin infections

a. Water provision

1. An adequate supply of water for washing and cleaning will reduce the burden of impetigo and scabies (**GRADE 2C**).^91^
2. From studies in remote Australian Indigenous communities, the installation of community swimming pools may assist in the prevention impetigo, along with other health benefits (**GRADE 2C**).^92-94^
3. No studies assessed the effect of quality water supply or swimming pools on scabies or tinea on which to base relevant recommendations for resource limited settings.

b. Housing improvement programs

1. Programs to improve housing may assist in the prevention and control of skin infections in resource-limited populations (**GRADE 2C**).^95,96^

**Supplementary Table 1:** List of studies included in the systematic review.

BA- Before and After; CBA- Controlled Before and After; CCT- Controlled clinical trial; CPS- Controlled Population Study; CRCT- Cluster Randomised Controlled Trial; ES- Ecological Study; PC- Prospective Cohort; RC-Retrospective Cohort; RCT- Randomised Controlled Trial

| **Study** | **Title** | **Study design** | **Study setting** | **Country** | **Number of participants/ population size** | **Condition** |
| --- | --- | --- | --- | --- | --- | --- |
| Bowen 2014^26^ | Short-course oral co-trimoxazole versus intramuscular benzathine benzylpenicillin for impetigo in a highly endemic region: an open-label, randomised, controlled, non-inferiority trial | RCT | remote Aboriginal communities in NT | Australia | 508 | impetigo |
| Carapetis 1995^92^ | Skin sores in Aboriginal children. | BA | remote Aboriginal community in NT | Australia | 81 | impetigo |
| Faye 2007^28^ | Oral amoxicillin vs. oral erythromycin in the treatment of pyoderma in Bamako, Mali: an open randomized trial | RCT | dermatology outpatient department | Mali | 132 | impetigo |
| Luby 2002^30^ | The effect of antibacterial soap on impetigo incidence, Karachi, Pakistan | CRCT | squatter settlements in urban area | Pakistan | 241 | impetigo |
| Luby 2005^31^ | Effect of handwashing on child health: a randomised controlled trial | RCT | squatter settlements in urban area | Pakistan | 600 households | impetigo |
| Nicolle 1990^29^ | Outcome following therapy of group A streptococcal infection in schoolchildren in isolated northern communities | RCT | Indigenous communities in Canada-1) Inuit and 2) Indigenous | Canada | 34 | impetigo |
| Tong 2010^27^ | Trimethoprim-sulfamethoxazole compared with benzathine penicillin for treatment of impetigo in Aboriginal children: a pilot randomised controlled trial | RCT | remote Aboriginal community in NT | Australia | 13 | impetigo |
| Alipour 2015^46^ | The efficacy of oral ivermectin vs. sulfur 10% ointment for the treatment of scabies. | RCT | dermatology outpatient department | Iran | 420 | scabies |
| Avila-Romay 1991^62^ | Therapeutic efficacy, secondary effects, and patient acceptability of 10% sulfur in either pork fat or cold cream for the treatment of scabies | RCT | orphanage | Mexico | 111 | scabies |
| Brooks 2002^47^ | Ivermectin is better than benzyl benzoate for childhood scabies in developing countries | RCT | hospital outpatients in tropical country | Vanuatu | 110 | scabies |
| Chhaiya 2012^32^ | Comparative efficacy and safety of topical permethrin, topical ivermectin, and oral ivermectin in patients of uncomplicated scabies | RCT | dermatology outpatient department | India | 315 | scabies |
| Garcia 2007^64^ | Use of ivermectin to treat an institutional outbreak of scabies in a low-resource setting | PC | hospital inpatients | Peru | 46 | scabies |
| Goldust 2012^41^ | Treatment of scabies: comparison of permethrin 5% versus ivermectin | RCT | dermatology outpatient department | Iran | 242 | scabies |
| Goldust 2013a^44^ | Ivermectin vs. lindane in the treatment of scabies | RCT | dermatology outpatient department | Iran | 440 | scabies |
| Goldust 2013b^33^ | Treatment of scabies: the topical ivermectin vs. permethrin 2.5% cream | RCT | dermatology outpatient department | Iran | 380 | scabies |
| Goldust 2013c^36^ | Comparison of permethrin 2.5% cream vs. Tenutex emulsion for the treatment of scabies | RCT | dermatology outpatient department | Iran | 440 | scabies |
| Goldust 2014^37^ | Topical ivermectin versus crotamiton cream 10% for the treatment of scabies | RCT | dermatology outpatient department | Iran | 340 | scabies |
| Haar 2014^50^ | Scabies community prevalence and mass drug administration in two Fijian villages | CPS | remote villages in tropics | Fiji | 760 | scabies |
| Kanaaneh 1976^65^ | The eradication of a large scabies outbreak using community-wide health education | PC | Arabic village community | Israel | 2902 | scabies |
| LaVincente 2009^63^ | Community management of endemic scabies in remote aboriginal communities of northern Australia: low treatment uptake and high ongoing acquisition. | PC | remote Aboriginal communities in NT | Australia | 596 (40 households) | scabies |
| Ly 2009^48^ | Ivermectin versus benzyl benzoate applied once or twice to treat human scabies in Dakar, Senegal: a randomized controlled trial | RCT | dermatology outpatient department | Senegal | 133 | scabies |
| Mapar 2008^43^ | The comparison of oral ivermectin and topical Lindane in the treatment of scabies | RCT | dermatology outpatient department | Iran | 57 | scabies |
| Mohammed 2012^51^ | Soil transmitted helminths and scabies in Zanzibar, Tanzania following mass drug administration for lymphatic filariasis - a rapid assessment methodology to assess impact | RC | primary health care centres in island districts | Tanzania | 50 health care units | scabies |
| Mohebbipour 2013^45^ | Comparison of oral ivermectin vs. lindane lotion 1% for the treatment of scabies | RCT | dermatology outpatient department | Iran | 148 | scabies |
| Mytton 2007^38^ | Safety of benzyl benzoate lotion and permethrin in pregnancy: a retrospective matched cohort study. | RC | refugee camps | Burma (Myanmar) | 640 | scabies |
| Oyelami 2009^105^ | Preliminary study of effectiveness of Aloe vera in scabies treatment | RCT | institutional- welfare and prison units | Nigeria | 30 | scabies |
| Pourhasan 2013^35^ | Treatment of scabies, permethrin 5% cream vs. crotamiton 10% cream | RCT | dermatology outpatient department | Iran | 350 | scabies |
| Ranjkesh 2013^42^ | The efficacy of permethrin 5% vs. oral ivermectin for the treatment of scabies | RCT | dermatology outpatient department | Iran | 68 | scabies |
| Abedin 2007^52^ | Efficacy of permethrin cream and oral ivermectin in treatment of scabies | CCT | urban boys hostel | India | 84 | scabies |
| Sharma R 2011^40^ | Topical permethrin and oral ivermectin in the management of scabies: a prospective, randomized, double blind, controlled study. | RCT | dermatology outpatient department | India | 120 | scabies |
| Sule 2007^49^ | Comparison of ivermectin and benzyl benzoate lotion for scabies in Nigerian patients | RCT | dermatology outpatient department | Nigeria | 210 | scabies |
| Sungkar 2014^39^ | Effectiveness of permethrin standard and modified methods in scabies treatment | RCT | Islamic boarding school | Indonesia | 46 | scabies |
| Agrawal 2012^53^ | Mass scabies management in an orphanage of rural community: an experience | PC | institutional- orphanage | India | 50 | scabies |
| Talukder 2013^22^ | Controlling scabies in madrasahs (Islamic religious schools) in Bangladesh | CRCT | Male Islamic religious boarding schools | Bangladesh | 164 | scabies |
| Worth 2012^106^ | Acute morbidity associated with scabies and other ectoparasitoses rapidly improves after treatment with ivermectin | PC | urban slums | Brazil | 81 | scabies |
| Zargari 2006^34^ | Comparison of the efficacy of topical 1% lindane vs 5% permethrin in scabies: a randomized, double-blind study | RCT | dermatology outpatient department | Iran | 117 | scabies |
| Andrews 2009^54^ | A regional initiative to reduce skin infections amongst aboriginal children living in remote communities of the Northern Territory, Australia. | ES | remote Aboriginal communities in NT | Australia | 2329 | scabies and impetigo |
| Carapetis 1997^55^ | Success of a scabies control program in an Australian aboriginal community | ES | remote island Aboriginal community | Australia | 200-250 | scabies and impetigo |
| Kearns 2015^58^ | Impact of an Ivermectin Mass Drug Administration on Scabies Prevalence in a Remote Australian Aboriginal Community. | PC | remote island Aboriginal community | Australia | 1013 | scabies and impetigo |
| Lawrence 2005^59^ | Control of scabies, skin sores and haematuria in children in the Solomon Islands: Another role for ivermectin | CPS | remote island villages | Solomon Islands | 261 | scabies and impetigo |
| Marks 2015^60^ | Long Term Control of Scabies Fifteen Years after an Intensive Treatment Programme. | PC | remote islands | Solomon Islands | 338 | scabies and impetigo |
| Romani 2015^57^ | Mass Drug Administration for Scabies Control in a Population with Endemic Disease. | CRCT | remote island communities in Fiji | Fiji | 2051 | scabies and impetigo |
| Ryder 1985^91^ | The childhood health effects of an improved water supply system on a remote Panamanian island | CPS | remote islands | Panama | 393 | impetigo and scabies |
| Shelby-James 2002^61^ | Impact of single dose azithromycin on group A streptococci in the upper respiratory tract and skin of Aboriginal children. | CBA | remote Aboriginal community in NT | Australia | 103 | Impetigo and scabies |
| Taplin 1991^56^ | Community control of scabies: a model based on use of permethrin cream | PC | remote Island Kuna Indian population | Panama | 756 | scabies and impetigo |
| Wong 2001^23^ | Outcome of an interventional program for scabies in an Indigenous community. | ES | remote Aboriginal community in NT | Australia | 217 | scabies and impetigo |
| Wong 2002^24^ | Factors supporting sustainability of a community-based scabies control program | PC | remote Aboriginal community in Top End NT | Australia | 217 | scabies and impetigo |
| Davis 2013^66^ | A novel clinical grading scale to guide the management of crusted scabies | RC | hospital inpatients in top end NT | Australia | 49 | crusted scabies |
| Huffam 1998^67^ | Ivermectin for Sarcoptes scabiei hyperinfestation | PC | Aboriginal hospital inpatients in Top End NT | Australia | 22 | crusted scabies |
| Lokuge 2014^68^ | Crusted scabies in remote Australia, a new way forward: lessons and outcomes from the East Arnhem Scabies Control Program | CBA | remote Aboriginal communities in Top End NT | Australia | 7 | crusted scabies |
| Chander 2012^71^ | Comparative evaluation of griseofulvin, terbinafine and fluconazole in the treatment of tinea capitis | CCT | dermatology outpatient department | India | 75 | tinea capitis |
| Deng 2011^72^ | A random comparative study of terbinafine versus griseofulvin in patients with tinea capitis in Western China | CRCT | Chinese ethnic population, near afghan border | China | 73 | tinea capitis |
| Foster 2005^69^ | A randomized controlled trial assessing the efficacy of fluconazole in the treatment of pediatric tinea capitis | RCT | multiple centres, unclear on setting type | United States, Guatemala, Chile, Costa Rica, India | 880 | tinea capitis |
| Ladan 2005^70^ | Comparison of the efficacy of fluconazole and griseofulvin in tinea capitis | RCT | dermatology outpatient department | Iran | 40 | tinea capitis |
| Sabzghabaee 2009^107^ | Safety and efficacy of terbinafine in a pediatric Iranian cohort of patients with tinea capitis | PC | dermatology outpatient department | Iran | 60 | tinea capitis |
| Amit 2013^77^ | A comparative study of mycological efficacy of terbinafine and fluconazole in patients of Tinea corporis | RCT | dermatology outpatient department | India | 116 | tinea corporis |
| Choudhary 2013^80^ | Efficacy and safety of terbinafine hydrochloride 1% cream vs. sertaconazole nitrate 2% cream in tinea corporis and tinea cruris: a comparative therapeutic trial | RCT | dermatology outpatient department | India | 38 | tinea corporis |
| Ghaninejad 2009^73^ | Sertaconazole 2% cream vs. miconazole 2% cream for cutaneous mycoses: a double-blind clinical trial. | RCT | dermatology outpatient department | Iran | 100 | tinea corporis |
| Jerajani 2013^79^ | Comparative assessment of the efficacy and safety of sertaconazole (2%) cream versus terbinafine cream (1%) versus luliconazole (1%) cream in patients with dermatophytoses: a pilot study | RCT | dermatology outpatient department | India | 83 | tinea corporis |
| Lakshmi 2013^81^ | Clinical efficacy of topical terbinafine versus topical luliconazole in treatment of tinea corporis/tinea cruris patients | RCT | dermatology outpatient department | India | 60 | tinea corporis |
| Manasi 2011^78^ | Comparative evaluation of effectivity and safety of topical amorolfine and clotrimazole in the treatment of tinea corporis | RCT | dermatology outpatient department | India | 99 | tinea corporis |
| Sharma A 2011^74^ | Efficacy and tolerability of sertaconazole nitrate 2% cream vs. miconazole in patients with cutaneous dermatophytosis | RCT | dermatology outpatient department | India | 260 | tinea corporis |
| Singal 2005^75^ | Comparative efficacy of topical 1% butenafine and 1% clotrimazole in tinea cruris and tinea corporis: A randomized, double-blind trial | RCT | dermatology outpatient department | India | 80 | tinea corporis |
| Thaker 2013a^82^ | A comparative study to evaluate efficacy, safety and cost-effectiveness between Whitfield's ointment+oral fluconazole versus topical 1% butenafine in tinea infections of skin | RCT | dermatology outpatient department | India | 120 | tinea corporis |
| Thaker 2013b^76^ | A comparative randomized open label study to evaluate efficacy, safety and cost effectiveness between topical 2% sertaconazole and topical 1% butenafine in tinea infections of skin | RCT | dermatology outpatient department | India | 125 | Tinea corporis |
| Vishalkshi 2015^108^ | An observational, comparative study to assess the efficacy and safety of topical clotrimazole cream 1% and miconazole gel 2% in dermatophytoses in real life clinical practice | CCT | two clinical settings, further details not provided | India | 255 | Tinea corporis |
| Chuku 2006^89^ | Control of Tinea (ringworm) using Aloe vera gel in Rivers State | PC | Boy's school | Nigeria | 45 | tinea capitis and tinea corporis |
| Dinkela 2007^90^ | Efficacy of triclosan soap against superficial dermatomycoses: a double-blind clinical trial in 224 primary school-children in Kilombero District, Morogoro Region, Tanzania. (Special issue: Global theme issue on poverty and health development.) | RCT | Schools | Tanzania | 52 | tinea capitis and tinea corporis |
| Koh 2003^83^ | Use of terbinafine for tinea in Australian Aboriginal communities in the Top End | PC | remote Aboriginal communities in NT | Australia | 44 | tinea corporis and tinea unguium |
| Amit 2007^84^ | An open randomized comparative study to test the efficacy and safety of oral terbinafine pulse as a monotherapy and in combination with topical ciclopirox olamine 8% or topical amorolfine hydrochloride 5% in the treatment of onychomycosis | RCT | dermatology outpatient department | India | 96 | onychomycosis |
| Bassiri-Jahromi 2012^87^ | A comparative evaluation of combination therapy of fluconazole 1% and urea 40% compared with fluconazole 1% alone in a nail lacquer for treatment of onychomycosis: therapeutic trial | RCT | unclear | Iran | 90 | onychomycosis |
| Grover 2007^109^ | Combination of surgical avulsion and topical therapy for single nail onychomycosis: a randomized controlled trial | RCT | dermatology outpatient department | India | 40 | onychomycosis |
| Pravesh 2015^85^ | Comparative efficacy of continuous and pulse dose terbinafine regimes in toenail dermatophytosis: a randomized double-blind trial | RCT | dermatology outpatient department | India | 76 | onychomycosis |
| Souza 2014^88^ | Randomized controlled trial comparing photodynamic therapy based on methylene blue dye and fluconazole for toenail onychomycosis | RCT | dermatology outpatient department | Brazil | 142 | onychomycosis |
| Succi 2013^86^ | Intermittent therapy with terbinafine and nail abrasion for dermatophyte toe onychomycosis: a pilot study | RCT | dermatology outpatient department | Brazil | 36 | onychomycosis |
| Aboriginal Environmental Health Unit 2010^96^ | Closing the gap: 10 Years of Housing for Health in NSW. An evaluation of a healthy housing intervention | CBA | Aboriginal communities in rural NSW | Australia | unclear | skin infections |
| Bailie 2012^95^ | The impact of housing improvement and socio-environmental factors on common childhood illnesses: a cohort study in Indigenous Australian communities. | PC | remote Aboriginal communities in NT | Australia | 10 communities | skin infections |
| Silva 2008^94^ | Effect of swimming pools on antibiotic use and clinic attendance for infections in two Aboriginal communities in Western Australia. | RC | remote Aboriginal communities in WA | Australia | 259 | skin infections |
| Lehmann 2003^93^ | Benefits of swimming pools in two remote Aboriginal communities in Western Australia: intervention study | BA | remote Aboriginal communities in WA | Australia | 121 | impetigo, fungal skin infections, scabies |
| Schmeller 2001^25^ | Skin diseases in children in rural Kenya: long-term results of a dermatology project within the primary health care system. | ES | schools and day care centres | Kenya | 5780 | impetigo, tinea capitis, tinea corporis, scabies |
| Oladele 2010^110^ | Management of superficial fungal infections with Senna alata ("alata") soap: a preliminary report | RCT | institutional-male prison setting | Nigeria | 23 | Tinea corporis, scabies, other fungal skin infections |

**Supplementary Table 2:** Risk of bias table with overall quality ratings using the GRADE approach for included experimental and controlled studies

+=low risk of bias (shaded orange), x=high risk of bias (shaded red),?=unclear risk of bias (shaded yellow)

CBA- Controlled Before and After; CCT- Controlled clinical trial; CPS- Controlled Population Study; CRCT- Cluster Randomised Controlled Trial; RCT- Randomised Controlled Trial

| **Study** | **Design** | **Random sequence generation** | **Allocation concealment** | **Blinding of personnel and participants** | **Blinding of outcome assessors** | **Incomplete outcome data** | **Selective outcome reporting** | **Other sources of bias** | **Quality Rating** |
| --- | --- | --- | --- | --- | --- | --- | --- | --- | --- |
| Alipour 2015 | RCT | ? | x | x | + | x | x | + | Moderate (B) |
| Amit 2007 | RCT | + | ? | x | ? | ? | + | + | Moderate (B) |
| Amit 2013 | RCT | ? | ? | x | x | ? | x | + | Low (C) |
| Avila-Romay 1991 | RCT | ? | ? | ? | + | + | + | ? | Moderate (B) |
| Bassiri-Jahromi 2012 | RCT | ? | ? | + | + | x | ? | + | Low (C) |
| Bowen 2014 | RCT | + | + | x | + | + | + | + | High (A) |
| Brooks 2002 | RCT | + | ? | x | + | + | + | + | High (A) |
| Chhaiya 2012 | RCT | + | x | x | ? | + | + | + | Moderate (B) |
| Choudhary 2013 | RCT | ? | x | x | x | x | ? | + | Very low (D) |
| Dinkela 2007 | RCT | ? | ? | ? | ? | x | x | x | Low (C) |
| Faye 2007 | RCT | + | ? | x | x | + | x | + | Moderate (B) |
| Foster 2005 | RCT | + | + | x | + | + | + | + | High (A) |
| Ghaninejad 2009 | RCT | ? | ? | + | + | x | + | + | Moderate (B) |
| Goldust 2012 | RCT | ? | ? | x | + | + | x | ? | Low (C) |
| Goldust 2013a | RCT | ? | ? | + | + | x | + | + | High (A) |
| Goldust 2013b | RCT | ? | ? | x | + | x | + | + | Moderate (B) |
| Goldust 2013c | RCT | ? | x | x | + | + | x | ? | Low (C) |
| Goldust 2014 | RCT | ? | ? | x | + | x | x | + | Low (C) |
| Grover 2007 | RCT | x | x | x | ? | x | x | x | Very low (D) |
| Jerajani 2013 | RCT | ? | ? | x | x | x | ? | x | Very low (D) |
| Ladan 2005 | RCT | ? | x | x | + | x | + | + | Low (C) |
| Lakshmi 2013 | RCT | x | x | x | x | x | ? | + | Very low (D) |
| Luby 2005 | RCT | + | + | + | + | + | + | ? | High (A) |
| Ly 2009 | RCT | + | ? | x | + | + | + | + | Moderate (B) |
| Manasi 2011 | RCT | ? | x | x | + | x | + | + | Low (C) |
| Mapar 2008 | RCT | ? | x | x | x | + | x | x | Moderate (B) |
| Mohebbipour 2013 | RCT | ? | ? | x | + | + | + | + | High (A) |
| Nicolle 1990 | RCT | + | ? | x | ? | x | x | + | Low (C) |
| Oladele 2010 | RCT | ? | x | x | x | ? | ? | ? | Low (C) |
| Oyelami 2009 | RCT | ? | x | x | x | + | ? | + | Low (C) |
| Pourhasan 2013 | RCT | ? | ? | x | + | ? | x | + | Low (C) |
| Pravesh 2015 | RCT | + | + | + | + | + | + | + | High (A) |
| Ranjkesh 2013 | RCT | ? | ? | x | + | ? | ? | ? | Low (C) |
| Sharma R 2011 | RCT | + | + | + | + | + | + | + | High (A) |
| Sharma A 2011 | RCT | ? | ? | + | + | + | + | + | High (A) |
| Singal 2005 | RCT | ? | + | + | + | x | + | x | Moderate (B) |
| Souza 2014 | RCT | ? | x | + | x | x | ? | ? | Low (C) |
| Succi 2013 | RCT | ? | x | x | x | ? | + | + | Low (C) |
| Sule 2007 | RCT | ? | x | x | x | ? | x | + | Moderate (B) |
| Sungkar 2014 | RCT | ? | x | + | x | x | + | x | Low (C) |
| Thaker 2013a | RCT | ? | x | x | x | x | + | + | Low (C) |
| Thaker 2013b | RCT | ? | x | x | x | x | + | + | Low (C) |
| Tong 2010 | RCT | + | + | x | + | + | + | + | High (A) |
| Zargari 2006 | RCT | ? | + | + | + | ? | + | + | High (A) |
| Deng 2011 | CRCT | ? | ? | x | x | + | + | x | Low (C) |
| Luby 2002 | CRCT | ? | x | + | ? | + | + | + | Moderate (B) |
| Romani 2015 | CRCT | + | ? | x | x | + | ? | + | Moderate (B) |
| Talukder 2013 | CRCT | ? | ? | x | + | + | + | ? | Moderate (B) |
| Aboriginal Environmental Health Unit 2010 | CBA | ? | x | x | ? | ? | + | + | Low (C) |
| Lokuge 2014 | CBA | x | x | x | x | x | x | ? | Very low (D) |
| Shelby-James 2002 | CBA | x | x | x | x | x | + | x | Very low (D) |
| Chander 2012 | CCT | x | x | x | ? | x | x | x | Very low (D) |
| Chuku 2006 | CCT | ? | x | x | x | x | ? | + | Very low (D) |
| Abedin 2007 | CCT | x | x | x | x | x | ? | x | Very low (D) |
| Haar 2014 | CPS | x | x | x | x | x | + | + | Moderate (B) |
| Lawrence 2005 | CPS | x | x | x | x | ? | x | + | Low (C) |
| Ryder 1985 | CPS | ? | x | ? | ? | ? | + | + | Low (C) |

**Supplementary Table 3:** Risk of bias table with overall quality rating using the GRADE approach for included observational studies

+=low risk of bias (shaded green), x=high risk of bias (shaded red),?=unclear risk of bias (shaded yellow)

BA- Before and After; ES- Ecological Study; PC- Prospective Cohort; RC-Retrospective Cohort

| **Author Year** | **Design** | **Differences between groups and selection bias** | **Measurement bias** | **Incomplete outcome data** | **Selective outcome reporting** | **Other sources of bias and confounding** | **Quality Rating** |
| --- | --- | --- | --- | --- | --- | --- | --- |
| Bailie 2012 | PC | x | x | x | + | x | Low (C) |
| Garcia 2007 | PC | + | x | x | + | x | Low (C) |
| Huffam 1998 | PC | + | x | + | + | + | Moderate (B) |
| Kanaaneh 1976 | PC | + | x | + | ? | + | Moderate (B) |
| Kearns 2015 | PC | + | x | + | + | ? | Low (C) |
| Koh 2003 | PC | + | x | x | + | ? | Low (C) |
| LaVincente 2009 | PC | x | x | x | ? | ? | Very low (D) |
| Marks 2015 | PC | ? | x | x | + | + | Low (C) |
| Sabzghabaee 2009 | PC | + | x | x | + | x | Very low (D) |
| Agrawal 2012 | PC | + | x | + | ? | + | Moderate (B) |
| Taplin 1991 | PC | + | x | x | ? | + | Low (C) |
| Wong 2002 | PC | ? | x | ? | x | + | Low (C) |
| Worth 2012 | PC | + | x | + | + | + | Moderate (B) |
| Davis 2013 | RC | x | x | + | + | ? | Low (C) |
| Mohammed 2012 | RC | ? | x | x | ? | x | Low (C) |
| Mytton 2007 | RC | + | x | x | + | + | Low (C) |
| Silva 2008 | RC | x | x | x | ? | x | Very low (D) |
| Vishalkshi 2015 | RC | x | x | + | ? | ? | Low (C) |
| Carapetis 1995 | BA | x | ? | x | + | + | Low (C) |
| Lehmann 2003 | BA | x | x | ? | ? | ? | Very low (D) |
| Andrews 2009 | ES | x | x | x | + | x | Low (C) |
| Carapetis 1997 | ES | ? | ? | ? | + | + | Low (C) |
| Schmeller 2001 | ES | + | x | ? | + | + | Moderate (B) |
| Wong 2001 | ES | x | x | ? | x | + | Low (C) |

**Supplementary Table 4.** Method of application of topical scabicides in 29 included studies

*Authors may have explained application as “whole body” or “all over the body”, however, this could mean sparing of any or all of the head, face or mucous membranes, and therefore was not considered of adequate detail to classify as head to toe or neck to toe

| **Study** | **Name of topical scabicide** | **Method of application** | | |
| --- | --- | --- | --- | --- |
|  |  | **Child cases** | **Adult cases** | **Contacts** |
| Alipour 2015 | Sulphur | Not specified | Not specified | Not specified |
| Andrews 2009 | Permethrin | Not specified* | Not applicable | Not specified |
| Brooks 2002 | Benzyl Benzoate | Not specified | Not specified | Not specified |
| Carapetis 1997 | Permethrin | Head to toe | Neck to toe | Adult contacts neck to toe |
| Chhaiya 2012 | Permethrin | Neck to toe | Neck to toe | Not applicable |
|  | Ivermectin | To affected sites | To affected sites | Not applicable |
| Goldust 2012 | Permethrin | Head to toe | Head to toe | Head to toe |
| Goldust 2013a | Lindane | Head to toe | Neck to toe | Not specified |
| Goldust 2013b | Permethrin | Neck to toe | Neck to toe | Not specified |
|  | Ivermectin | To affected sites | To affected sites | Not specified |
| Goldust 2013c | Permethrin | Head to toe | Neck to toe | Not specified |
|  | Tenutex (disulfuram and benzyl benzoate) | Head to toe | Neck to toe | Not specified |
| Goldust 2014 | Crotamiton | Not specified | Not specified | Not specified |
|  | Ivermectin | To affected sites | To affected sites | Not specified |
| Haar 2014 | Benzyl benzoate | Neck to toe | Neck to toe | Not specified |
| Kanaaneh 1976 | Benzyl benzoate | Not specified | Not specified | Not specified |
| LaVincente 2009 | Permethrin | Not specified* | Not specified* | Not specified* |
| Ly 2009 | Benzyl Benzoate | Neck to toe | Neck to toe | Not specified |
| Mapar 2008 | Lindane | Head to toe | Head to toe | Not specified |
| Mohebbipour 2013 | Lindane | Not specified | Not specified | Not specified |
| Mytton 2007 | Permethrin | Not applicable | Neck to toe | Not applicable |
|  | Benzyl Benzoate | Not applicable | Neck to toe | Not applicable |
| Pourhasan 2013 | Permethrin | Head to toe | Neck to toe | Not specified |
|  | Crotamiton | Head to toe | Neck to toe | Not specified |
| Ranjkesh 2013 | Permethrin | Neck to toe | Neck to toe | Not specified |
| Romani 2015 | Permethrin | Neck to toe | Neck to toe | Neck to toe |
| Abedin 2007 | Permethrin | Neck to toe | Not applicable | Not applicable |
| Sharma R 2011 | Permethrin | Neck to toe | Neck to toe | Not specified |
| Sule 2007 | Benzyl Benzoate plus tetraethylthiuram monosulfine | Neck to toe | Neck to toe | Not specified |
| Sungkar 2014 | Standard application of permethrin | Neck to toe | Neck to toe | –Not applicable |
|  | Modified application of permethrin | To affected sites | To affected sites | Not applicable |
| Agrawal 2012 | Benzyl Benzoate | Neck to toe | Not applicable | Not applicable |
| Talukder 2013 | Permethrin | Neck to toe | Not applicable | Neck to toe |
| Taplin 1991 | Permethrin | Head to toe | Head to toe | Not specified |
| Wong 2001 | Permethrin | Head to toe | Not applicable | Not specified |
| Zargari 2006 | Permethrin | Head to toe | Head to toe | Not specified |
|  | Lindane | Head to toe | Head to toe | Not specified |
